# Impact of the *MIF* -173G/C variant on cardiovascular disease risk: a meta-analysis of 9,047 participants

**DOI:** 10.3389/fcvm.2024.1323423

**Published:** 2024-02-26

**Authors:** Hamas Fouda, Wisam N. Ibrahim, Zumin Shi, Fahad Alahmadi, Yousef Almohammadi, Amal Al-Haidose, Atiyeh M. Abdallah

**Affiliations:** ^1^ Department of Biomedical Sciences, College of Health Sciences, QU Health, Qatar University, Doha, Qatar; ^2^ Human Nutrition Department, College of Health Sciences, QU Health, Qatar University, Doha, Qatar; ^3^ Pediatric Department, College of Medicine, Taibah University, King Faisal Specialist Hospital, Al-Madinah, Saudi Arabia; ^4^ Pediatric Department, Security Forces Medical Centre, Al-Madinah, Saudi Arabia

**Keywords:** cardiovascular disease, macrophage migration inhibitory factor, polymorphism, meta-analysis, Arab, Asian, European

## Abstract

**Introduction:**

Many factors contribute to the risk of cardiovascular disease (CVD), an umbrella term for several different heart diseases, including inflammation. Macrophage migration inhibitory factor (MIF) is an important immune modulator that has been shown to be involved in the pathogenesis of different heart diseases, so understanding pathogenic variants of the *MIF* gene is important for risk stratification. We therefore conducted a meta-analysis to investigate whether the *MIF* -173G/C (rs755622) polymorphism is associated with CVD.

**Methods:**

The PubMed, Science Direct, and Embase databases were searched from inception to June 2023 for case-control studies of the *MIF* -173G/C polymorphism and its relationship to any type of CVD. Correlations between the *MIF* -173G/C polymorphism and CVD were estimated by pooling the odds ratios (ORs) with 95% confidence intervals in allelic, dominant, and recessive models using random-effects meta-analysis.

**Results:**

A total of 9,047 participants (4141 CVD cases and 4906 healthy controls) from 11 relevant studies were included. In the total population, there was no significant association between the *MIF* -173G/C (rs755622) polymorphism and the risk of developing CVD in the three different models. In a stratified analysis by ethnicity, the allelic model (C vs G) was significantly associated with CVD in the Arab and Asian populations (OR = 0.56, CI 0.42 -0.75 and OR = 1.28, CI 1.12 -1.46, respectively); the dominant model (CC+CG vs GG) was significantly associated with CVD in the Arab population (OR = 0.42, CI 0.30 -0.61); while the recessive model (GG+GC vs CC) was associated with CVD susceptibility in the Arab population (OR = 3.84, CI 1.57 -9.41). There were no significant associations between the *MIF* -173 G/C polymorphism and CVD risk in the European population. Conclusion, the *MIF* -173G/C polymorphism is associated with CVD in some populations.

**Systematic Review Registration:**

https://www.crd.york.ac.uk/PROSPERO/, PROSPERO (CRD42023441139).

## 1 Introduction

Macrophage migration inhibitory factor (MIF) is an immune cytokine with pro-inflammatory, enzymatic, and hormonal functions implicated the pathogenesis of inflammatory and neoplastic diseases. MIF has various functions including in leukocyte recruitment, regulation of immune responses, inflammation, counter-regulation of glucocorticoid activity, and cellular proliferation (1). It is expressed in several immune cell types including T cells, neutrophils, monocytes/macrophages, and eosinophils and also in pituitary cells, epithelial cells, smooth muscle cells, and cardiomyocytes (2, 3), suggesting that it can have diverse roles in various pathophysiological processes (4, 5). MIF is known to play a critical role in both innate and acquired immune responses and it upregulates the expression of pro-inflammatory cytokines (6, 7). In addition, MIF is implicated in cardiovascular diseases (CVD), acting as a reliable biomarker of disease severity and being readily detectable in the blood and at sites of inflammation (8). MIF may therefore have significant impact on the prognosis of CVD patients through its ability to modulate the disease phenotype.

CVD is a common and leading cause of mortality and morbidity worldwide (9, 10). Recognizing CVD as a serious concern for global health, the WHO launched the 25 × 25 Action Plan in 2013 to reduce premature mortality due to non-communicable diseases by 25% by 2025 (11). The 2015 Global Burden of Diseases study estimated that there are 422.7 million CVD cases and 17.59 million CVD deaths worldwide (10). Furthermore, its prevalence is increasing, mostly due to population growth and aging populations, with especially high prevalences in South and East Asia due to their large and rapidly growing populations. Conversely, CVD mortality rates decreased by ∼15% between 1990 and 2015 in some high-income and some middle-income countries (11), while mortality rates have plateaued in high-income regions such as Western Europe, North America, and Australia. Overall, middle-to-low-income countries appear to be disproportionately burdened by CVD mortality. Additionally, CVD occurs approximately 7–10 years later in females than in males, although it remains a major cause of death in females over 65 years. For instance, recent data from the National Health and Nutrition Examination revealed that the prevalence of MI has increased in females aged between 35 and 45 years over the past two decades while decreasing in similarly aged males (12). Deaths due to CVD are most amenable to rapid intervention, and preventing deaths from CVD requires reliable data on CVD risk factors to inform effective treatment and prevention. Individual predisposition to CVD is determined by both environmental and genetic risk factors, the most prevalent environmental factors being hypertension, hypercholesteremia, diabetes, obesity, smoking, stress, gender, ethnic origin, and a sedentary lifestyle (13–15).

**Figure 1:**
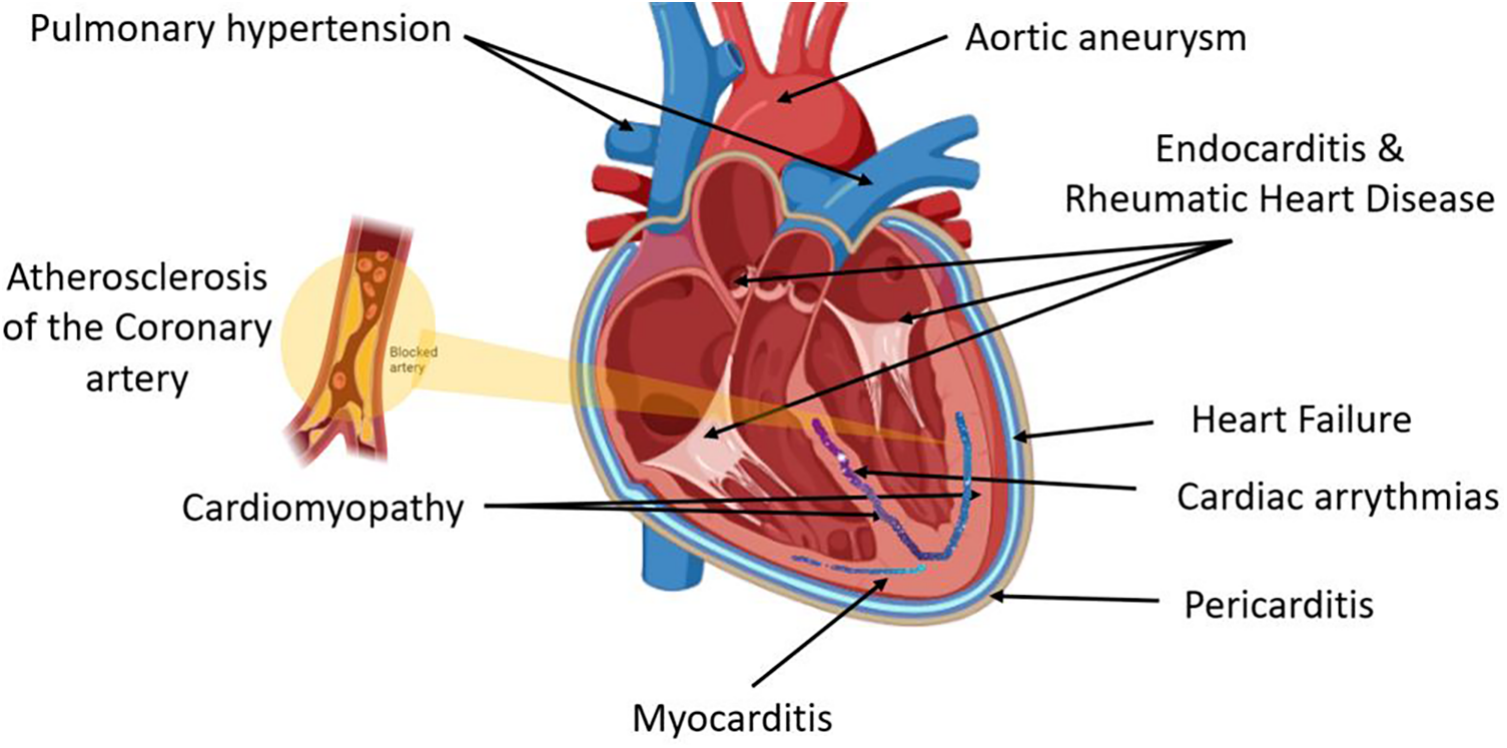
Cardiovascular disease (CVD) is an umbrella term for different heart diseases that include abnormalities of the pericardial layer (e.g., pericarditis and pericardial effusion), myocardial layer [e.g., myocarditis, cardiomyopathy, coronary artery disease (CAD), myocardial infarction (MI), heart failure (HF), coronary artery abnormalities (CAA), acute coronary syndrome (ACS)], the endocardial layer [e.g., valvular heart diseases including endocarditis and rheumatic heart disease (RHD)], abnormalities of the cardiac conductive system including all cardiac arrythmias, and lastly arterial abnormalities such as hypertension and aneurysm.

CVD is an umbrella term for different heart diseases including coronary artery disease (CAD), myocardial infarction (MI), heart failure (HF), coronary artery abnormalities (CAA), acute coronary syndrome (ACS), and rheumatic heart disease (RHD) (Figure 1). CHD is defined as the narrowing of the coronary arteries leading to a reduced luminal diameter and hence a decrease in blood flow, and it is the most common cause of MI (16). Hypertension and hypercholesteremia accelerate atherosclerotic plaque development and formation due to endothelial injury, which increases endothelial permeability and allows plasma components to infiltrate (17). Plaque hemorrhage ultimately activates thrombus formation initiated by platelet aggregation. Furthermore, cholesterol and triglycerides contribute to plaque formation, both positively and negatively (18). Prolonged and silent atherosclerosis and plaque formation involve chronic inflammatory reactions that influence immune cell, platelet, and complement recruitment to the site of injury (18). Recent compelling evidence highlights a central role for macrophage proliferation within atherosclerotic lesions in driving disease progression. These macrophages, initially immune cells, undergo multiplication, contributing significantly to the pool of foam cells within arterial walls (19). As part of this process, myocardium-produced MIF is highly associated with the development of various CVDs (20), and there is increasing evidence that MIF plays a major role in atheroma formation and CVD progression.

*MIF* is a short gene (<0.7 kb) at 22q11.2 composed of three short exons of 107, 172, and 66 base pairs (21). The *MIF* promoter harbors two polymorphisms that have a regulatory effect on gene transcription (22): the -974 CATT tetranucleotide repeat, which exists in 5–8 repeats (rs5844572), and the -173 G-to-C polymorphism (rs755622). The CATT_5_ repeat is associated with low *MIF* expression compared with the CATT_6_, CATT_7_, and CATT_8_ repeat alleles. By contrast, -173C allele is associated with high *MIF* gene expression (23). Both polymorphisms have been reported to be associated with different autoimmune and inflammatory diseases. A meta-analysis of 23 articles from different populations representing 5,559 cases and 7,335 controls reported an association between the -173G/C polymorphism and susceptibility to a wide range of different autoimmune diseases (24). Karakaya et al. reported an association between the *MIF* -173C allele and erythema nodosum in Löfgren syndrome patients but not sarcoidosis, indicating a role for MIF after the sarcoid inflammatory response has begun (25). Interestingly, MIF demonstrated a specific role in the recruitment and accumulation of inflammatory macrophages in an animal model of polymicrobial sepsis (26). Moreover, MIF was found to play an important role as a stress molecule counteracting the immunosuppressive effect of glucocorticoids in renal inflammation (27), and MIF deficiency suppressed apoptosis and protected the liver from ischemia-reperfusion injury (28). Conversely, MIF has been shown to play a protective role in Parkinson's disease (29).

Therefore, there is evidence that MIF is associated with CVD, with an association between the *MIF* -173C/G polymorphism and CVD reported in some but not all populations. To clarify this association, here we conducted a meta-analysis based on a systematic literature review to confirm whether *MIF* -173G/C (rs755622) is associated with the risk of developing CVD.

## 2 Materials and methods

### 2.1 Study design and objectives

**Figure 2:**
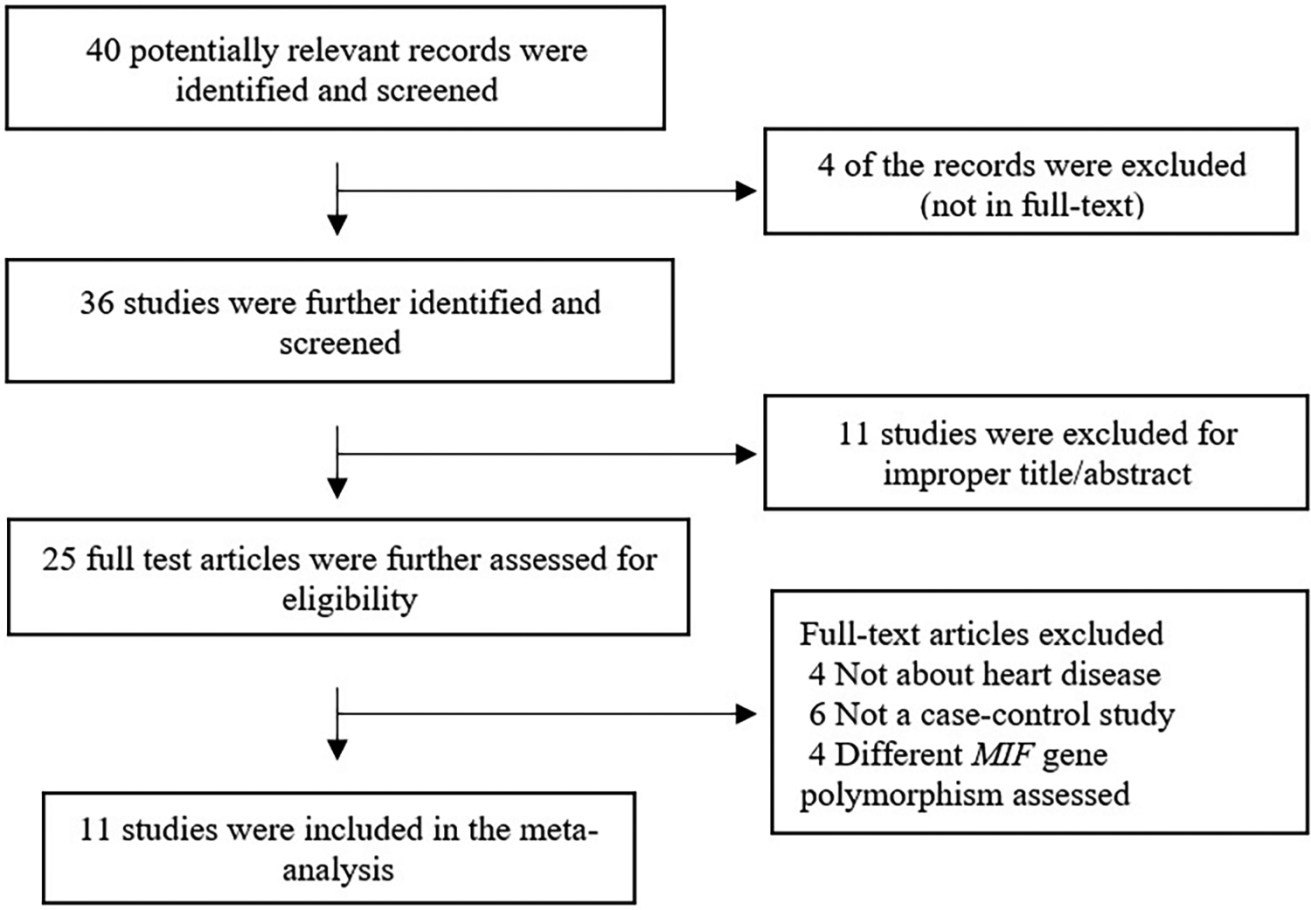
Flow chart of the literature review.

This review followed Preferred Reporting Items for Systematic Review and Meta-Analysis (PRISMA) guidelines (Figure 2) (30) and was registered in the International Prospective Register of Systematic Reviews (PROSPERO; CRD42023441139) database. A PICO strategy was used to guide the study design: population, patients with CVD; intervention, association between the *MIF* -173G/C variant and CVD; and primary outcome, the association between *MIF* -173G/C and CVD.

### 2.2 Search strategy

The PubMed, Science Direct, and Embase databases were searched from inception of these databases to June 2023 using the following terms: macrophage migration inhibitory factor or *MIF* [TEXT WORD] and cardiovascular disease or coronary artery disease [TEXT WORD] or cardiomyopathy [TEXT WORD] or cardiac surgery [TEXT WORD] or HF [TEXT WORD] or rheumatic heart disease [TEXT WORD] or kawasaki disease [TEXT WORD], MI [TEXT WORD] or intracoronary thrombosis [TEXT WORD] or acute coronary syndrome [TEXT WORD] or sudden cardiac death [TEXT WORD]. In addition, the reference lists of compatible articles and conferences were reviewed. Two authors individually screened each article by title and abstract and then evaluated the full text to fully assess eligibility for inclusion.

### 2.3 Inclusion and exclusion criteria

The inclusion criteria were: (1) all conditions affecting the heart or blood vessels were included as CVD; (2) evaluated *MIF* -173 G/C polymorphisms and cardiovascular risk; (3) case-control or nested case-control design; (4) included the genotypes for the *MIF* -173G/C gene polymorphism in CVD cases and controls; and (5) the study reported that the distribution of genotypes among controls was in Hardy-Weinberg equilibrium (HWE). Exclusion criteria were: (1) failed to provide detailed data in the abstract and review; (2) the study was a duplicate; (3) failed to report the genotype frequency; and (4) the controls failed to meet HWE.

### 2.4 Data extraction and models

The author's details, date of publication, region of study, population ethnicity, number of genotypes analyzed, and the total number of cases and controls were recorded from each article. Two authors individually extracted the required data for each study article, and any disagreement was resolved by consensus or by consultation with a senior author.

Three different genetic models were used to assess the association between the genetic variant and the outcome: the dominant model (Model 1) was defined as the presence of the common allele (CC + CG vs. GG); the recessive genetic model (Model 2) was the presence of rare allele (GG + GC vs. CC); and the allelic model assessed the association between the alleles (C vs. G) and the outcome, regardless of whether it was dominant or recessive.

### 2.5 Quality assessment

Quality was assessed using the Newcastle-Ottawa quality assessment scale (NOS) for case-control studies. Data quality was judged based on comparability, selection, and outcome of interest for case-control study articles and was noted using a “star system”. To compare study quality, star counts were totaled (Table 1). Data validity was assessed by senior authors based on the provided criteria.

**Table 1:** Characteristics of studies included in the meta-analysis.

No	Reference	Country	Ethnicity	CVD type	Case number	Control number	Genotyping	Newcastle-Ottawa score
1	Idouz et al. 2019 (32)	Morocco	Arab	Dilated cardiomyopathy (DCM)	53	50	TaqMan	6/7
2	El-Mahdy et al. 2021 (33)	Egypt	Arab	Heart failure	90	60	PCR-RFLP	6/7
3	Abdallah et al. 2016 (34)	Saudi Arabia	Arab	Rheumatic heart disease	124	202	TaqMan	6/7
4	Simonini et al. 2008 (35)	Italy	European	Kawasaki disease	69	60	PCR-RFLP	6/7
5	Tereshchenko et al. 2009 (36)	Czech	European	Myocardial infarction	219	137	TaqMan	5/7
	Tereshchenko et al. 2009 (36)	Russian	European	Myocardial infarction	240	174	PCR-SSP	
6	Luo et al. 2016 (37)	Chinese Kazakh	Asian	Coronary artery disease	320	603	TaqMan	4/7
7	Ji et al. 2015 (38)	Chinese Han	Asian	Coronary heart disease	70	186	PCR	3/7
8	Luo et al. 2021 (39)	Chinese Han	Asian	Coronary artery disease	1,176	1,120	TaqMan	5/7
9	Du et al. 2020 (40)	Chinese	Asian	Acute coronary syndrome	699	1,153	TaqMan	5/7
10	Zhang et al. 2022 (41)	Chinese	Asian	Acute coronary syndrome	963	932	50-Plex SNPscan	6/7
11	Qian & Ripeng 2018 (31)	Chinese	Asian	Coronary heart disease	118	229	PCR-RFLP	3/7
				Total	4,141	4,906		

### 2.6 Statistical analysis

All statistical analyses were performed in STATA v17 (StataCorp, College Station, TX, United States). Heterogeneity between studies was assessed with the I^2^ statistic. The pooled odds ratio (OR) with 95% CI in the forest plot was analyzed using a random-effects model [restricted maximum likelihood (REML) method] with the subgroup option in Stata. Begg's funnel plot was used to qualitatively assess the risk of publication bias. All analyses were performed using Stata 18. A *p*-value <0.05 (two-sided) was considered statistically significant in all analyses.

## 3 Results

### 3.1 Study characteristics

Figure 1 summarizes the search process. Forty articles were found in the initial search, 29 of which were excluded after applying exclusion criteria. Eleven articles met the inclusion criteria and were used in the meta-analysis, representing 4,906 controls and 4,141 cases.

The characteristics of the included articles are summarized in Table 1. Of the included studies, three were performed in Arab populations, six in Asian populations, and two in European populations (31–41). All included studies were cross-sectional case-control studies that included the necessary data to calculate the possible association between the *MIF* -173G/C polymorphism and CVD. One study was published in Chinese, while the remaining studies were published in English.

The individual studies' quality was appraised utilizing the Newcastle-Ottawa Scale (NOS) scoring system. According to the NOS results, 73% of the included studies achieved a score of 5 or higher out of 7 on the NOS scale, indicating an overall good level of quality (Table 1).

### 3.2 Quantitative data synthesis

**Figure 3:**
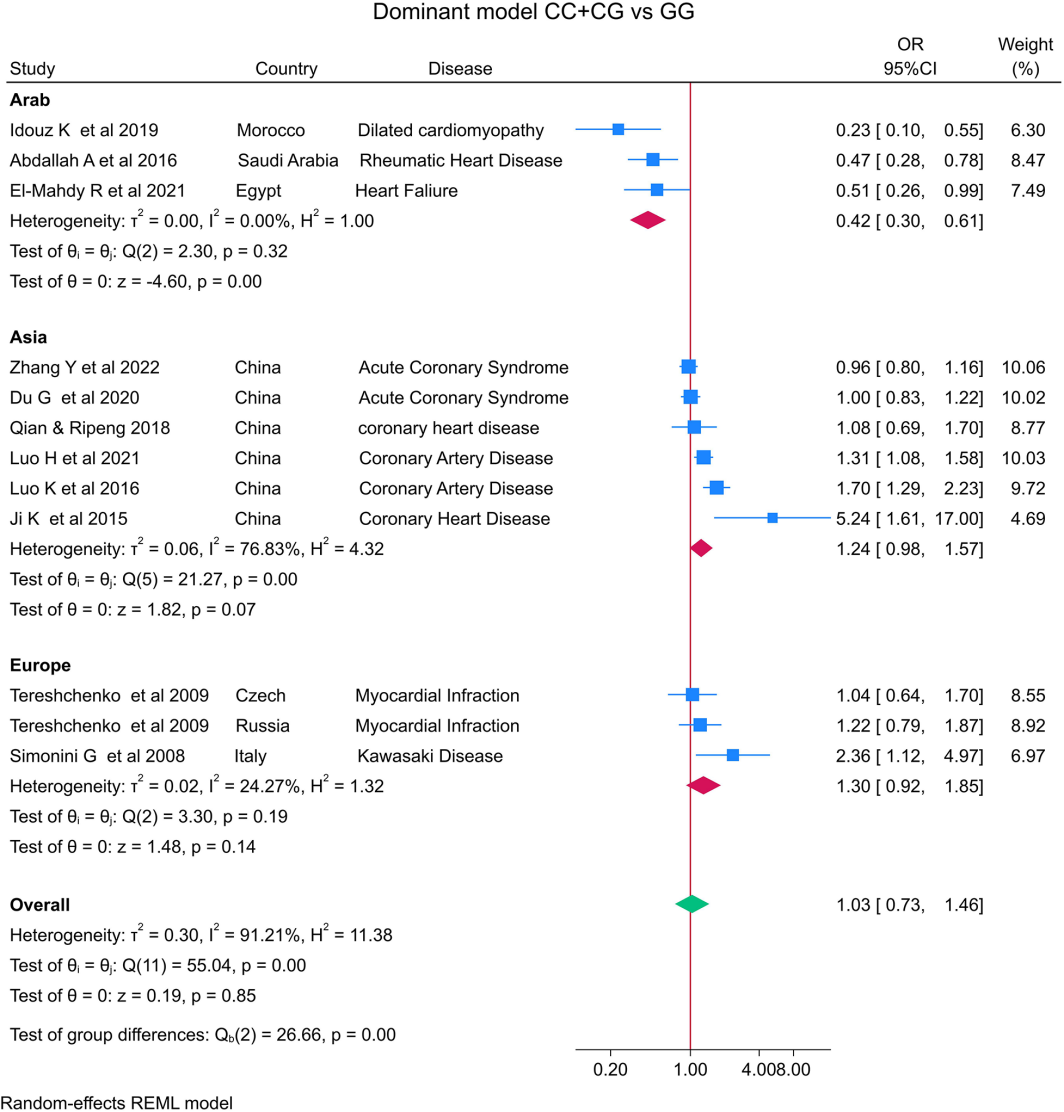
Forest plot of cardiovascular disease risk associated with the *MIF* -173 G/C polymorphism in Model 1 (CC + CG vs. GG) in Arab, Asian, and European ethnicities.

**Figure 4:**
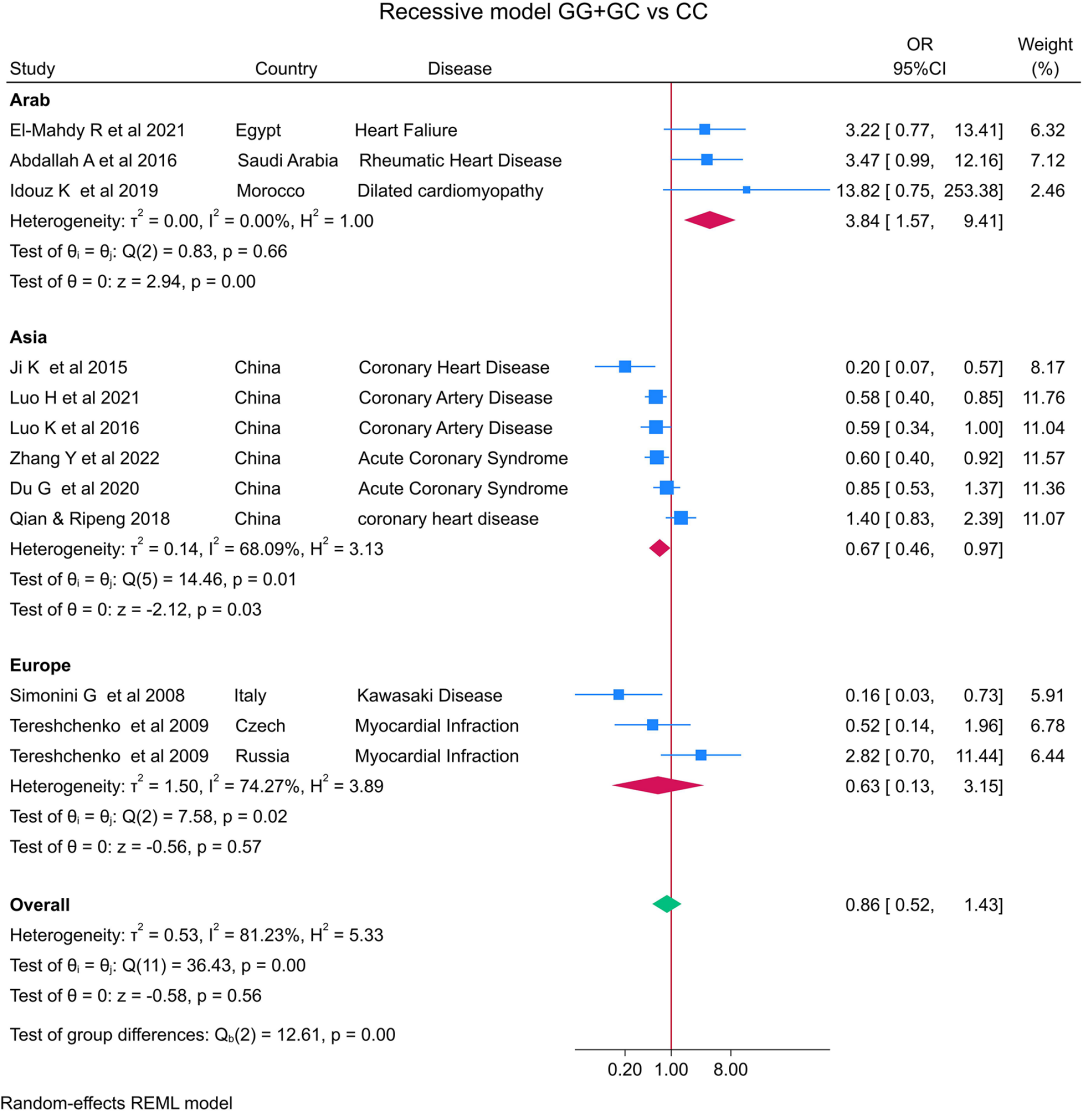
Forest plot of cardiovascular disease risk associated with the *MIF* -173 G/C polymorphism in model 2 (GG + GC vs. CC) in Arab, Asian, and European ethnicities.

**Figure 5:**
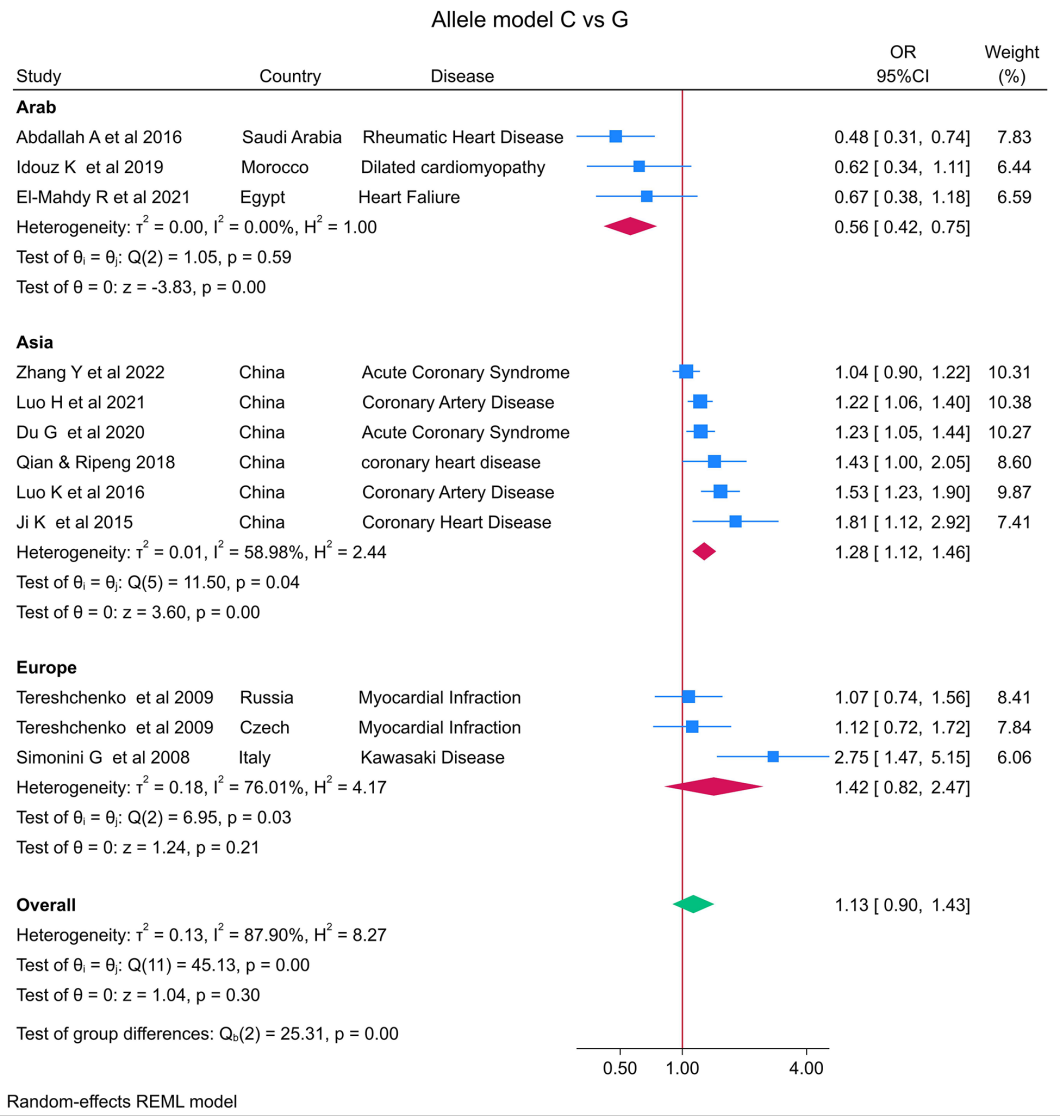
Forest plot of cardiovascular disease risk associated with the *MIF* -173 G/C polymorphism in model 3 (C vs. G) alleles in Arab, Asian, and European ethnicities.

The distribution of the *MIF* -173 genotype in CVD is shown in Table 2, and the meta-analysis results are shown in Table 3. There was no significant impact of the *MIF* -173G/C polymorphism and the risk of CVD in the three models assessed: Model 1 (dominant): CC + CG vs. GG (Figure 3), Model 2 (recessive): GG + GC vs. CC (Figure 4), and Model 3 (allelic): C vs. G (Figure 5).

**Table 2:** Genotypes and allele frequencies *of MIF* -173G/C genes in CVD patients and controls.

Study	Case					Control						
	GG	GC	CC	G	C	GG	GC	CC	G	C	Sample size	HWE (P)
Idouz et al. 2019 (32)	29	18	6	76	30	11	39	0	61	39	53/50	0.01
El-Mahdy et al. 2021 (33)	51	36	3	87	39	24	30	6	54	36	90/60	0.15
Abdallah et al. 2016 (34)	95	26	3	216	32	122	64	16	308	96	124/202	0.07
Simonini et al. 2008 (35)	46	19	14	111	47	46	12	2	104	16	69/60	0.30
Tereshchenko et al. 2009 (36)	163	47	9	373	65	103	31	3	237	37	219/137	0.71
Tereshchenko et al. 2009 (36)	164	73	3	401	79	126	42	6	294	54	240/174	0.30
Luo et al. 2016 (37)	153	140	27	446	194	367	205	31	939	267	320/603	0.73
Ji et al. 2015 (38)	46	14	10	106	34	136	44	6	316	56	70/186	0.31
Luo et al. 2021 (39)	688	411	77	1,787	565	703	373	44	1,779	461	1,176/1,120	0.53
Du et al. 2020 (40)	396	272	31	1,064	334	727	382	44	1,836	470	699/1,153	0.48
Zhang et al. 2022 (41)	586	317	60	1,489	437	559	337	36	1,455	409	963/932	0.09
Qian & Ripeng 2018 (31)	71	26	21	168	68	142	73	14	357	101	118/229	0.27

HWE, Hardy-Weinberg equilibrium; MIF, macrophage migration inhibitory factor; CVD, cardiovascular disease.

**Table 3:** Summary of different meta-analysis results.

Study	Sample size			Test of association			Heterogeneity		
	Case	Control	Number of studies	OR (95% CI)	Z	*p*-value	*X* ^2^	*p*-value	*I*^2^ (%)
CC + CG vs. GG									
Overall	4,279	5,069	12	1.03 [0.73 to 1.46]	0.19	0.85	0.30	0.00	91.2
Arab	267	312	3	0.42 [0.30 to 0.61]	−4.60	0.00	0.00	0.32	0.00
European	528	371	3	1.30 [0.92 to 1.85]	1.48	0.14	0.02	0.19	24.2
Asian	3,484	4,386	6	1.24 [0.98 to 1.57]	1.82	0.07	0.06	0.00	76.8
GG + GC vs. CC									
Overall	4,279	5,069	12	0.86 [0.52 to 1.43]	−0.58	0.56	0.53	0.00	81.2
Arab	267	312	3	3.84 [1.57 to 9.41]	2.94	0.00	0.00	0.66	0.00
European	528	371	3	0.63 [0.13 to 3.15]	−0.56	0.57	1.50	0.02	74.2
Asian	3,484	4,386	6	0.67 [0.46 to 0.97]	−2.12	0.03	0.14	0.01	68.1
C vs. G									
Overall	4,279	5,069	12	1.13 [0.90 to 1.43]	1.04	0.30	0.13	0.00	87.9
Arab	267	312	3	0.56 [0.42 to 0.75]	−3.83	0.00	0.00	0.59	0.0
European	528	371	3	1.42 [0.82 to 2.47]	1.24	0.21	0.18	0.03	76
Asian	3,484	4,386	6	1.28 [1.12 to 1.46]	3.60	0.00	0.01	0.04	59

In the stratified analysis by ethnicity, Model 1 (CC + CG vs. GG) and Model 2 (GG + GC vs. CC) demonstrated significant associations between the *MIF* -173G/C polymorphism and CVD in the Arab population (OR = 0.42, CI 0.30 to 0.61, *p* < 0.001 and OR = 3.84, CI 1.57 to 9.41, *p* < 0.001) (Figures 3, 4) but not in the European and Asian populations. Model 3 (C vs. G) also demonstrated a significant association between the *MIF* -173G/C polymorphism and the risk of CVD in the Arab population (OR = 0.56, CI 0.42 to 0.75, *p* < 0.001) (Figure 5) and in the Asian population (OR = 1.28, CI 1.12 to 1.46, *p* < 0.001) but not in the European population.

### 3.3 Publication bias analyses

**Figure 6:**
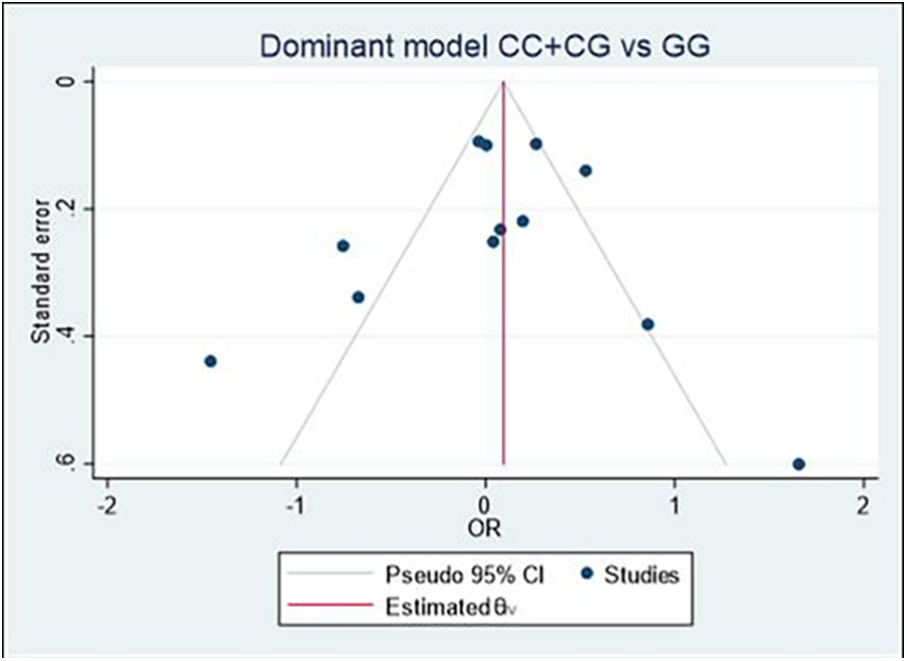
Begg's funnel plot of the MIF -173 polymorphism and CVD for all models; Model 1: CC + CG vs. GG, Model 2: GG + GC vs. CC, and Model 3: C vs. G. Egger's test was used to provide statistical evidence of funnel plot symmetry for the three models, and no evidence of publication bias was found in Model 1 (*p* = 0.851), Model 2 (*p* = 0.154), and Model 3 (*p* = 0.687).

Begg's funnel plot and Egger's test were performed to assess publication bias (Figure 6). For all three models, there was no obvious asymmetry and there was no evidence of publication bias for Model 1 (*p* = 0.851), Model 2 (*p* = 0.154), or Model 3 (*p* = 0.687).

## 4 Discussion

In this meta-analysis, we aimed to comprehensively review and quantify the literature to establish whether the *MIF* -173G/C (rs755622) polymorphism is associated with a risk of CVD development. By meta-analyzing eleven studies representing 4,279 cases and 5,069 controls, we found no significant association between the *MIF* -173G/C polymorphism and the risk of CVD in the overall study population in the three models assessed: CC + CG vs. GG (OR = 1.03), GG + GC vs. CC (OR = 0.86), and C vs. G allele (OR = 1.13). In addition, due to overall heterogeneity and variability in study outcomes between different studies, we conducted a subgroup analysis of the different ethnicities, which revealed that the *MIF* -173G/C polymorphism is significantly associated with a decreased risk of CVD in the Arab population but not the Asian or European populations in the CC + CG vs. GG model. In the second GG + GC vs. CC model, there was again a significant association between the *MIF* -173G/C polymorphism and the risk of CVD in the Arab population but not the Asian or European populations. Finally, for the C vs. G allele model, a significant association was observed in the Arab population (OR = 0.56) and the Asian population (OR = 1.28) but not the European population for the *MIF* -173G/C polymorphism and CVD risk. Our findings are similar to other meta-analyses of *MIF*, where the C allele was found to be more common within CAD patients (42) and those with chronic kidney diseases (43). However, in systemic lupus erythematosus, while serum MIF levels were associated with the disease, a meta-analysis found no association between the -173G/C polymorphism and the disease (44).

In atherosclerosis, macrophages play a pivotal role, undergoing proliferation and apoptosis. Macrophage proliferation contributes to plaque inflammation, while apoptosis, if excessive, may lead to plaque instability (45). MIF influences macrophage functions, promoting recruitment and inhibiting migration. This interplay is crucial in atherosclerotic plaque development. Within plaques, activated macrophages release pro-inflammatory signals and transform into foam cells by engulfing oxidized LDL cholesterol. The balance between macrophage dynamics and MIF's influence determines the progression and severity of atherosclerosis. Blockade of MIF reduces the aortic inflammatory response and is associated with reduction in aortic plaque and foam cell formation (46). In addition to its direct effects on inflammation and plaque stability, MIF interacts intricately with CXCL4L1, leading to the formation of prothrombotic and proinflammatory MIF-CXCL4L1 heterocomplexes (47, 48). These heterocomplexes have been implicated in promoting endothelial dysfunction, thrombosis, and exacerbation of inflammatory responses within the vascular environment (49). The presence of the -173 polymorphism in the MIF gene may modulate the formation or activity of these heterocomplexes, potentially influencing the progression and severity of CAD. Consequently, understanding the interplay between MIF protein levels, genetic variations, and the formation of MIF-CXCL4L1 heterocomplexes is crucial for deciphering the multifaceted molecular mechanisms underlying CAD and devising targeted therapeutic strategies aimed at disrupting these detrimental interactions (50).

Studies of patients with MI have demonstrated dual functions for the *MIF* polymorphism depending on disease severity and the patient's age. For instance, when cardiac ischemia is brief, *MIF* secreted by cardiomyocytes is cardioprotective through activation of AMP-activated protein kinase (AMPK) (51). Phosphorylation of AMPK stimulates glucose uptake through glucose transporter-4 (GLUT4). Conversely, when myocardial ischemia is prolonged, MIF activates immune cells, thereby increasing inflammation and cardiac remodeling by utilizing myofibroblasts to promote matrix protein synthesis (51). Similarly, Abdallah et al. (34) reported a similar dual function for *MIF* in RHD patients, with a lower frequency of the *MIF* -173C allele in RHD patients compared with controls and in those with later disease onset. Their findings suggested that MIF may help to clear pathogens and apoptotic cells during the early stages of RHD, perhaps protecting cardiomyocytes and delaying valvular damage. Conversely, after repetitive rheumatic insults, MIF may accelerate the recruitment of inflammatory cells and pro-inflammatory mediators, increasing regional inflammation and cardiac tissue damage. Similar studies on other diseases have also demonstrated that *MIF* is age-dependent. For instance, Das et al. (52) reported that adults expressing the low MIF (CATT_5_) allele were more susceptible to Gram-negative bacterial infections, while Lehmann et al. (53) found that adults expressing high levels of *MIF* polymorphisms were protected from sepsis mortality. In animal models, the protective role of MIF was lost in aged animals after ischemic heart injury, with low *MIF* expression impairing AMPK activation (54). These data suggest that it is important to consider the patient's age and disease stage when analyzing *MIF* polymorphisms.

Sex has also been reported to be associated with *MIF* polymorphisms. The MONICA/KORA Augsburg study concluded that female carriers of the *MIF* -173C polymorphism were at higher risk of coronary heart disease (55). This result was later confirmed in two studies of Chinese populations (37, 38). In inflammatory diseases, *MIF* -173 was found to be a disease severity marker for male multiple sclerosis patients (56), while the minor homozygous genotype for both the 974 CATT repeat and the -173G/C polymorphism were reported to protect female patients from major depressive disorder (57). However, another study showed that the *MIF* -173C allele is a susceptibility factor for depression in type 2 diabetes patients (58). These data suggest that these two polymorphisms are sex-specific disease modifiers.

There are several limitations to our meta-analysis. First, we identified relatively few studies for inclusion, and independent validation is now needed, especially for different ancestries. The number of studies for certain diseases and demographic subgroups was small, and the control group in one study was not in HWE. This precluded meaningful subgroup analysis with specific genotypes. In addition, several records without available original data were excluded from the final analysis. The chance of publication bias is high, as studies with statistically significant results are more likely to be published. The lack of representation of certain ethnicities leads to a reduction in the overall heterogeneity of the study samples, so the results require cautious interpretation. The influence of the MIF -173G/C variant on CVD may be affected by genetic, lifestyle, or environmental factors that were inconsistently measured across studies. This lack of consistent measurement may have led to underreporting of these phenomena in the context of MIF variants and CVD within the scope of this meta-analysis. Finally, variations in diagnostic methodology and criteria for CVD can contribute to inconsistencies, compromising the data integrity of published results. Consequently, these discrepancies may restrict the relevance of this meta-analysis.

In conclusion, our meta-analysis suggests that the *MIF* -173G/C polymorphism is not significantly associated with the risk of cardiovascular disease in the overall population. In subgroup analysis by ethnicity, the polymorphism was associated with a decreased risk of CVD in the Arab population. Future meta-analyses should consider the dual effect of *MIF* and the other promoter polymorphisms as well as disease status, sex, and patient age.

## Data Availability

The original contributions presented in the study are included in the article/Supplementary Material, further inquiries can be directed to the corresponding author.
